# Glomus Tumor of the Face: A Case Report and Literature Review

**DOI:** 10.1055/s-0045-1806765

**Published:** 2025-03-13

**Authors:** Sanjay Maurya, Sumedha Wadhwa, Onkar Singh, Peeyush Bhatt

**Affiliations:** 1 Army Hospital Research and Referral, New Delhi, India

**Keywords:** facial glomus tumor, magnetic resonance imaging, extradigital glomus tumor, A-V malformation

## Abstract

Glomus tumors are benign mesenchymal neoplasms arising from the glomus body. The most common site of presentation of a glomus tumor is the subungual region, followed by the fingertip and foot. Facial glomus tumors are extremely rare and constitute less than 1% of all glomus tumors. We report a case of facial glomus tumor that presented with a small painful nodule on the face. Following its excision, the diagnosis of glomus tumor came as a histopathological surprise.

## Introduction

Glomus tumors are benign mesenchymal tumors characterized by the proliferation of modified smooth muscles of the perivascular tissue called the glomus cells. They account for 1 to 2% of soft tissue tumors. 1 The most common presentation is a subungual nodule with the classic triad of symptoms: pain, localized tenderness, and cold hypersensitivity. However, extradigital glomus tumors are uncommon, with nonspecific signs and symptoms. The facial glomus tumors are particularly rare. 2 We report a case of a facial glomus tumor, which came as a histological surprise.

## Case Report

A 50-year-old woman presented with a nodular swelling of 18-month duration involving a small area about 2 cm inferior to the medial canthus of the right eye.

**Fig. 1:**
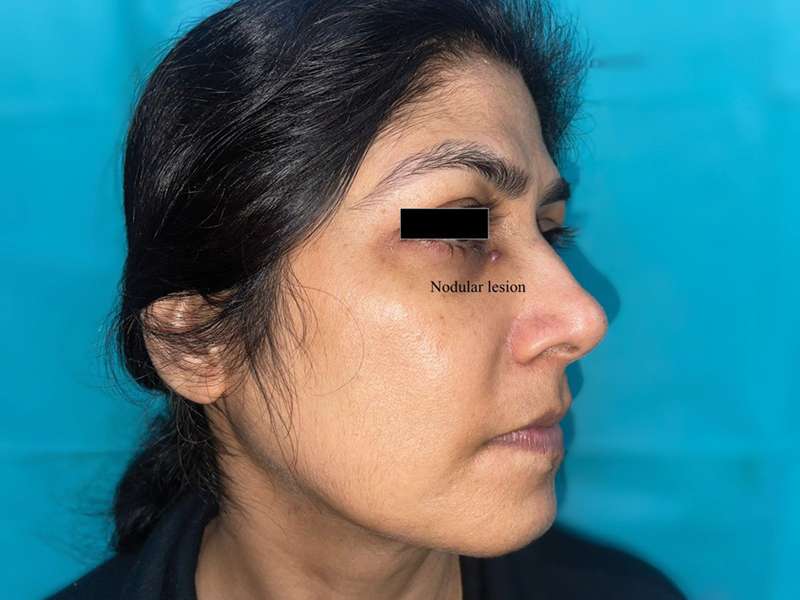
Small discolored nodule below the medial canthus.

The swelling was painless at its initial presentation. However, for the past 6 months, she noticed an increase in the size of the swelling, which was accompanied with pain on accidental touch and on wearing spectacles. There was also associated reddish discoloration of the swelling. There was no history of antecedent trauma or bleeding from the swelling. On examination, she was found to have a nodular lesion of 5 mm diameter over the inferior aspect of the medial canthus of the eye ( Fig. 1 ). The nodule was tender, hyperemic, and red. Adjacent to the lesion in the nasojugal groove, pulsatile flow was palpable.

**Fig. 2:**
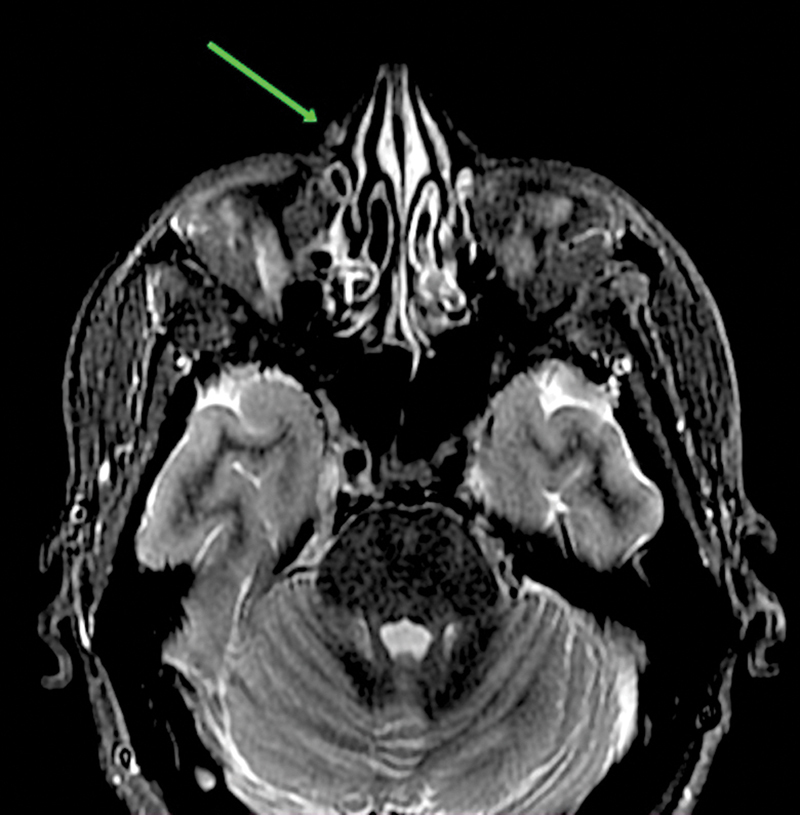
MRI showing altered signal intensity lesion and contrast-enhanced nodule in the right lateral nasal wall.

**Fig. 3:**
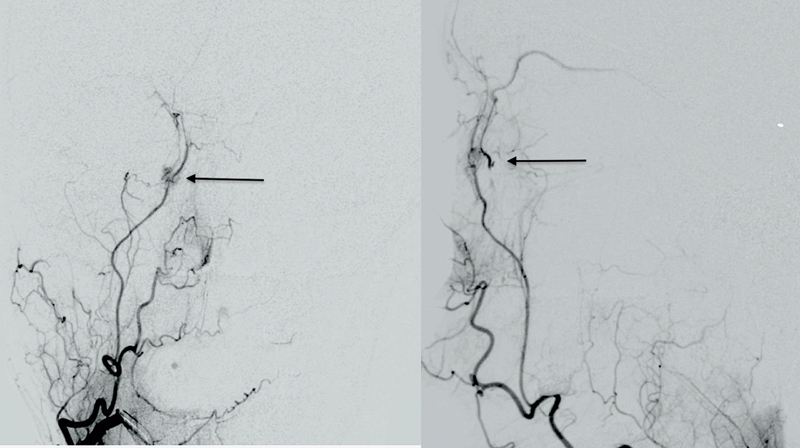
Digital subtraction angiography: Contrast blush in the angular branch of the facial artery around the right nasal wall with persistence of blush even after passage of dye beyond the nasal area.

Contrast-enhanced magnetic resonance imaging (MRI) revealed a small, altered signal intensity lesion in the subcutaneous plane overlying the right side of the nasal ridge measuring approximately 4 mm in maximum diameter. Few serpentine signal voids were seen in relation to the lesion. On postcontrast imaging, the lesion was seen in close relation to the superficial vessels of the face and showed postcontrast enhancement ( Fig. 2 ). The underlying right nasal bone and frontal process of the maxilla were normal in intensity and morphology. A preoperative digital subtraction angiography (DSA) was also done, which showed the persistence of contrast blush in the angular branch of the facial artery ( Fig. 3 ). The findings were suggestive of arteriovenous (AV) fistula.

**Fig. 4:**
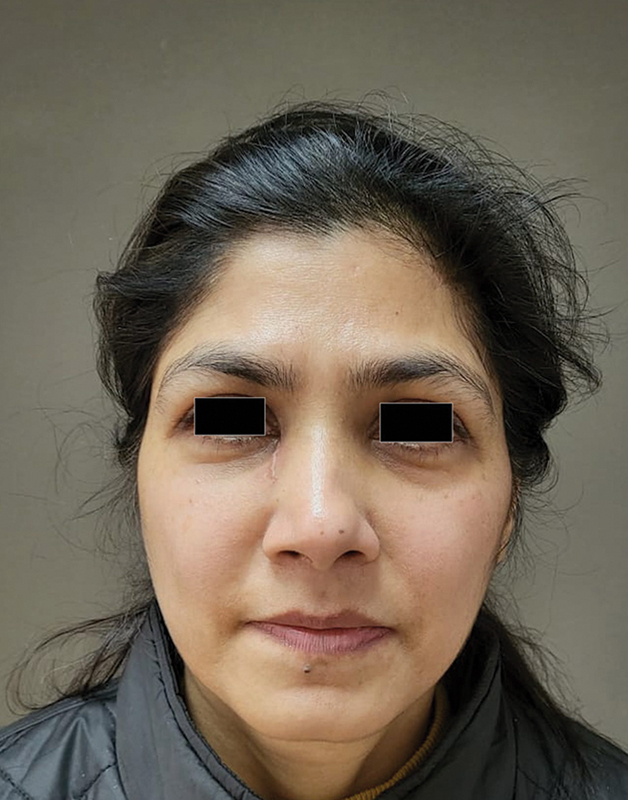
Postoperative status.

In view of the clinical and radiological findings, working diagnosis of AV fistula/vascular malformation was made. The patient was planned for a wide local excision of the lesion under general anesthesia under loupe magnification. An elliptical incision was marked approximately 3 mm from the lesion in such a way that primary closure was possible without distorting the lower eyelid in the nasal sidewall. The incision factored to the angular branch of the facial artery for vascular control. The artery was identified and clipped. Similarly, the draining vein on the superior aspect of the lesion was also identified and clipped. The lesion was completely excised with clear margins. Postoperative recovery was uneventful. At 3 months of follow-up, she remained asymptomatic without any features of recurrence ( Fig. 4 ). The histopathological diagnosis was of a glomus tumor with free margins.

**Fig. 5:**
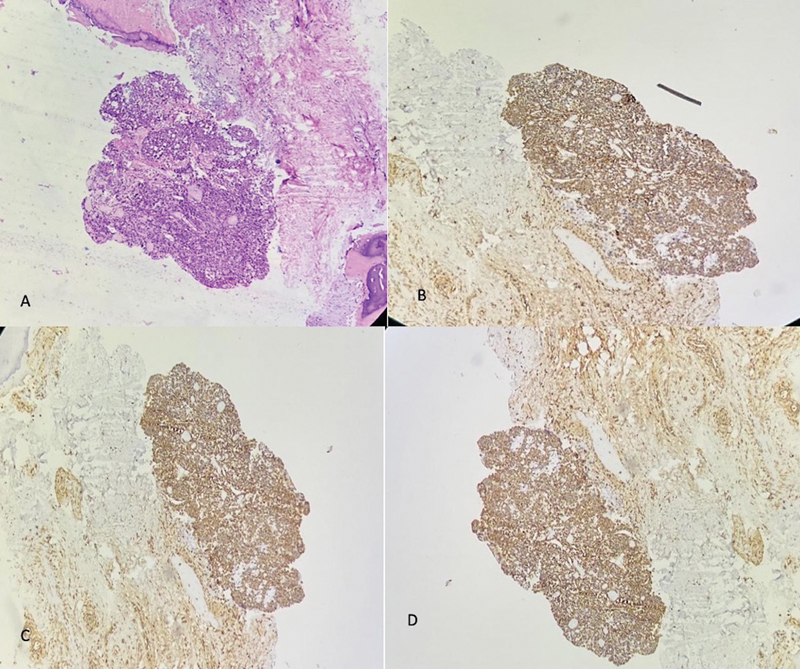
Histopathological examination and immunohistochemistry (IHC; 10x). ( **A** ) Hematoxylin and eosin (H&E) section. ( **B** ) Vimentin. ( **C** ) Calponin. ( **D** ) S100. Diagnosis of benign glomus tumor was confirmed on biopsy.

On immunohistochemistry, the tumor was positive for S100, calponin, and vimentin ( Fig. 5 ).

## Discussion

Glomus tumor is a benign vascular mesenchymal neoplasm composed of cells resembling modified smooth muscle cells of the normal glomus body. The glomus body, a thermoregulator, is a specialized form of AV anastomosis localized in dermal and precoccygeal soft tissue. These tumors comprise less than 2% of all soft tissue tumors. 3

There are two forms of glomus tumor, with the more common solitary variant accounting for most of the cases (90%), and a rarer multiple variant accounting for 10% of cases; this second form is seen most often in children and is thought to be inherited in an autosomal dominant fashion. 4

Depending on the predominant component, there are three histological variants of glomus tumors: (1) angiomatoid (glomangioma) with predominant blood vessels, (2) solid (poor vasculature and scant smooth muscle components), and (3) glomangiomyoma (predominantly smooth muscle). Glomangiomyoma is the least common histological variant of glomus tumor as described by Yang et al. 5 All these histological variants may be clinically indistinguishable. Ours was histologically classified as solid type, which is the commonest histological variant.

Painful nodules are the most common presentation of glomus tumors, occurring mostly in the subungual region, followed by the fingertips and feet. Extradigital glomus tumors are uncommon with nonspecific symptoms rendering them difficult to diagnose clinically. Amidst the various studies on extradigital glomus tumors, a retrospective review of extradigital glomus tumors was done at the Mayo clinic between 1985 and 2005. The study included 56 patients, of whom only 2 patients had facial glomus tumor—one on the nose and the other on the cheek. 6 Our case was difficult to diagnose preoperatively and came as a histological surprise. The clinical preoperative misdiagnosis of a glomus tumor in the form venous lake or epidermal cyst has also been reported by Wang et al. 7

MRI for preoperative radiological diagnosis is the gold standard. However, MRI can be suggestive of vascular lesions in the setting of a glomus tumor, a diagnosis that can be confirmed only on histopathology. Thus, histopathology is imperative in all cases undergoing excision. In our case, the MRI was suggestive of AV malformation and hence a DSA was done. It was the histology and immunohistochemistry that clinched our diagnosis. Hence, this case report highlights the rare nature of a facial glomus tumor and underscores the importance considering this diagnosis as incomplete excision can result in recurrence.

In conclusion, we reported the case of an extradigital glomus tumor arising below the medial canthus of the right eye and abutting the right lateral nasal wall. The lesion clinically appeared to be a vascular lesion, which was further confirmed with MRI. However, the histopathological report came as a surprise, suggesting the excised lesion to be a glomus tumor. Unusual tumor sites and differing clinical symptoms occasionally interfere with diagnosing and treating patients with extradigital tumors. Therefore, it is important to include the glomus tumor in the differential diagnosis of patients with extradigital painful or asymptomatic lesions with color variation.
